# IL-13 and IL-33 Serum Levels Are Increased in Systemic Sclerosis Patients With Interstitial Lung Disease

**DOI:** 10.3389/fmed.2022.825567

**Published:** 2022-02-17

**Authors:** Antonio Giovanni Versace, Alessandra Bitto, Carmelo Ioppolo, Caterina Oriana Aragona, Daniela La Rosa, William Neal Roberts, Tommaso D'Angelo, Antonella Cinquegrani, Santa Cirmi, Natasha Irrera, Michele Navarra, Salvatore Corrao, Sebastiano Gangemi, Gianluca Bagnato

**Affiliations:** ^1^Department of Clinical and Experimental Medicine, University of Messina, Messina, Italy; ^2^Department of Medicine, University of Kentucky, Lexington, KY, United States; ^3^Department of Biomedical Sciences and Morphological and Functional Imaging, University of Messina, Messina, Italy; ^4^Department of Pharmacy-Drug Sciences, University of Bari “Aldo Moro”, Bari, Italy; ^5^Department of Chemical, Biological, Pharmaceutical and Environmental Sciences, University of Messina, Messina, Italy; ^6^Department of Health Promotion Sciences, Maternal and Infant Care, Internal Medicine and Medical Specialties (PROMISE), University of Palermo, Palermo, Italy; ^7^Department of Internal Medicine, National Relevance and High Specialization Hospital Trust, Civico Di Cristina Benfratelli, Palermo, Italy

**Keywords:** systemic sclerosis, interstitial lung disease, IL-33, IL-13, interleukins

## Abstract

**Objective:**

Systemic sclerosis (SSc) mortality is extremely variable in its internal organ involvement. Pulmonary fibrosis occurs in up to 30% of the cases. Animal models provide evidence that IL-33 is able to induce both cutaneous and pulmonary fibrosis via increased IL-13 and in SSc patients the levels of IL-33 correlate with skin fibrosis. Our aim was to test whether both IL-33 and IL-13 are higher in patients with diffuse SSc and interstitial lung disease (SSc-ILD) compared to SSc patients without ILD and healthy controls.

**Methods:**

Serum levels of IL-13 and IL-33 were measured in 30 SSc patients with diffuse disease and 30 healthy controls by enzyme-linked immunosorbent assay. The extent of pulmonary fibrosis was assessed according to HRCT Warrick score. Pulmonary function tests included lung diffusion capacity for carbon monoxide, forced vital capacity and total lung capacity.

**Results:**

Both IL-13 and IL-33 levels were increased in SSc patients compared to controls and significantly associated each other. DLco, FVC and TLC scores were inversely associated with IL-33 and IL-13 levels. Both IL-33 and IL-13 levels were significantly associated with the Warrick severity score and higher in the group of SSc patients with reduced pulmonary function compared to SSc patients with normal pulmonary function tests.

**Conclusion:**

The IL-13/IL-33 axis needs to be further explored in longitudinal studies of SSc-ILD patients to assess its validity as a biomarker and future treatment target, as does downstream mediator ST2.

## Introduction

Systemic sclerosis (SSc) is a connective tissue disease characterized by the triad of microvascular injury, immunologic activation and fibrosis (1). The clinical phenotype of the disease differs widely. It is therefore crucial to apply strict patient selection criteria to identify effective treatments for life-threatening organ complications, thereby addressing one of the main clinical unmet need in SSc research (2). In up to 60% of SSc cases mortality is due to pulmonary involvement and half of these cases are related to the development of pulmonary fibrosis, with the other half due to pulmonary hypertension (3).

Although the pathogenesis of SSc needs to be clarified, previous studies confirm that some cytokines and growth factors influence fibrosis progression in SSc by stimulating the activation of fibroblasts, impairing endothelial cells activity, and altering immune system function (4, 5). We focused our attention in particular to IL-13 and IL-33, in light of the growing body of recent evidence suggesting a possible role for these cytokines in fibrogenesis.

IL-33 is one of the most recently discovered member of the IL-1 cytokine family (6–9), while IL-13 is a prototypical Th2 cytokine (10–12). Recent evidence show that the Th2 cytokines IL-4 and IL-13 are higher in SSc patients and promote fibrotic responses (12–14).

Animal models studies demonstrated that IL-33 is able to induce fibrosis via increased IL-13 both in cutaneous (15) and pulmonary (16) fibrosis. In addition, the level of IL-33 is correlated with the extent of skin fibrosis in SSc patients–being higher in patients with the diffuse form compared to those with limited SSc—as well as with forced vital capacity (FVC) scores (17–19). Furthermore, the polymorphism of IL-33 gene rs7044343 is associated with SSc susceptibility and pulmonary manifestations in different populations (20, 21).

Indeed, IL-33 and its receptor ST2 are abnormally expressed in SSc and it has been postulated that in early SSc, IL-33 could be mobilized from areas of vascular damage to promote fibrosis in target organ through ST2 (18, 22) and the differentiation of Treg lymphocytes toward a Th2-like phenotype (23).

Therefore, our aim was to verify the preliminary hypothesis that both IL-33 and IL-13 are higher in patients with diffuse SSc and interstitial lung disease (SSc-ILD) compared both to SSc patients without ILD, as measured through pulmonary function tests (DLco, TLC, FVC), and to healthy controls.

## Materials and Methods

### Patients

This is a single-center cross-sectional study involving SSc patients fulfilling 2013 criteria for SSc proposed by the American College of Rheumatology (24) and classified as diffuse form, according to the classification criteria of Le Roy et al. (25).

Among 90 eligible participants, those having pre-existing respiratory disorders and smokers (*n* = 7), SSc patients with the limited form (*n* = 47) and patients with overlap syndrome (*n* = 6) were excluded. Thus, 30 patients with the diffuse form of SSc were included in the study. Fourteen SSc patients were under treatment with immunosuppressants (11 with mycophenolate, mofetil, 9 with corticosteroids, 3 with azathioprine). Thirty age and sex-matched healthy individuals were included in the study as healthy controls.

### Clinical Assessment

Complete demographic and clinical profiles were collected for all participants at enrollement. The modified Rodnan skin score score (mRSS) was measured by summing skin thickness measurements on a scale of 0–3 in 17 body areas (26). The study was approved by the ethical committee of the University of Messina (protocol number 1/2016).

### Measurement of Serum IL-33 and IL-13

The serum concentrations of IL-33 and IL-13 levels were quantified using specific enzyme- linked immunosorbent assay (ELISA) kits (R&D Systems, Minneapolis, MN, USA), according to the manufacturer's indications.

### Pulmonary Function Assessment

All participants underwent pulmonary function measurements using a computerized spirometric system (Med Graphics Corporation; St. Paul, Minnesota, USA). Single-breath diffusing capacity for carbon monoxide (DL_CO_) was also measured and the values corrected for hemoglobin levels.

### Radiologic Assessment

Interstitial lung disease was defined as bibasilar interstitial fibrosis on high-resolution computed tomography (HRCT) of the chest. The presence of interstitial lung disease was evaluated by topographically dividing the lung into segments, using the Warrick score which is calculated according to the HRCT extent and appearance. The severity score ranges from normal (0) to all lesion present (15) while the extension score ranges from normal (0) to more than nine pulmonary segments involved (15). The sum of the severity and the extension scores represents the total Warrick score (27).

### Statistical Analysis

The Mann–Whitney U test was used to compare IL-33 and IL-13 levels. Fisher's exact probability test was used to compare frequencies, while the bivariate relationships between variables under study were assessed using the Spearman correlation coefficient. Linear regression analysis was used to examine the relationship between two variables. Multiple regression analysis were fitted according to the outcome of interest and analyzed separately due to the limited number of cases. The exploratory approach of the study allowed to analyze IL-13 and Il-33 as independent variables with a single dependent variable each time (DLco, TLC, FVC, HRCT Warrick severity score). A probability (*p*) value of < 0.05 was considered significant. All analyses were conducted using SPSS version 22 (SPSS, Inc., Chicago, IL, USA). Graphs were created using GraphPad Prism 6 (GraphPad Sofware, La Jolla CA, USA).

## Results

As shown in Table 1, 30 dcSSc patients participated in the study (26 women and 4 men; age 58.5 ± 12.4 years) and 30 healthy controls (25 women and 5 men; age 57.6 ± 13.5).

**Table 1:** Demographics and outcomes of interest of systemic sclerosis patients and controls.

	**dcSSc (***n*** = 30)**	**Controls (***n*** = 30)**	***p***
Age, mean ± SD years	58.5 ± 12.4	57.6 ± 13.5	0.78
Women, no. (%)	26 (87)	25 (83)	
Raynaud's phenomenon duration, mean ± SD years	8.8 ± 5	/	
Disease duration (onset of non-RP symptoms), mean ± SD years	5.6 ± 3.8	/	
IL-33 pg/ml, mean ± SD	36.8 ± 23.4	12.4 ± 8.6	<0.0001
IL-13 pg/ml, mean ± SD	0.84 ± 0.65	0.35 ± 0.18	0.0002
Warrick severity score, mean ± SD	4.6 ± 4.31	/	
Warrick extension score, mean ± SD	3.58 ± 3.03	/	
HRCT pulmonary fibrosis, no. (%)	16 (53)	/	
DLCo, mean ± SD	72 ± 19.9	/	
DLCo <70%, no. (%)	14 (46)	/	
TLC, mean ± SD	75.8 ± 16.5	/	
TLC <70%, no. (%)	11 (36)	/	
FVC, mean ± SD	78.5 ± 18.3	/	
FVC <70%„ no. (%)	11 (36)	/	
mRSS, mean ± SD	18.5 ± 6.2	/	
ANA+, no. (%)	30 (100)	/	
Scl70+, no. (%)	12 (40)	/	
ACA+, no. (%)	3 (10)	/	
RNA III+, no. (%)	3 (10)	/	
SRC, no (%)	0 (0)	/	
Costipation, no (%)	3 (10)	/	
Diarrhea, no (%)	2 (6)	/	
Gastritis, no (%)	7 (23)	/	
Proctitis, no (%)	2 (6)	/	
GERD, no (%)	19 (63)	/	

*The table shows the demographic features and the outcomes of interest of systemic sclerosis patients with diffuse cutaneous form (dcSSc) and healthy controls. No statistical differences were observed in gender or age between the groups while significantly higher concentrations of both IL-33 and IL-13 were found in dcSSc patients. Additionally, autoantibody profile, disease and Raynaud's phenomenon duration, modified Rodnan skin score (mRSS), pulmonary function tests and Warrick scores of scleroderma patients are reported*.

*dcSSc, diffuse cutaneous form of systemic sclerosis; RP, Raynaud's phenomenon; HRCT, high resolution computed tomography; DLco, diffusion capacity for carbon monoxide; FVC, forced vital capacity; TLC, total lung capacity; ANA, anti-nuclear antibody; Scl70, anti-Scl70 antibody; ACA, anti-centromere antibody; RNA III, anti-RNA polymerase III antibody; SRC, scleroderma renal crisis; GERD, gastroesophageal reflux disease*.

Anti-topoisomerase I (Scl70) antibodies were positive in 12 patients, anticentromere antibodies in 3, anti-RNA polymerase III (RNAIII) antibodies in 3. The median disease duration, calculated from the first non-Raynaud symptom was 5.6 ± 3.8 years.

### IL-13 and IL-33 Serum Levels and Disease Outcomes

First, we wanted to assess whether there was any difference in serum levels of IL-33 and IL-13 between SSc patients and controls. We found that IL-13 and IL-33 levels were significantly higher in SSc patients compared to controls. Furthermore, we observed that IL-13 and IL-33 are directly correlated with one another (*r*^2^ = 0.32, *p* = 0.0009).

**Figure 1:**
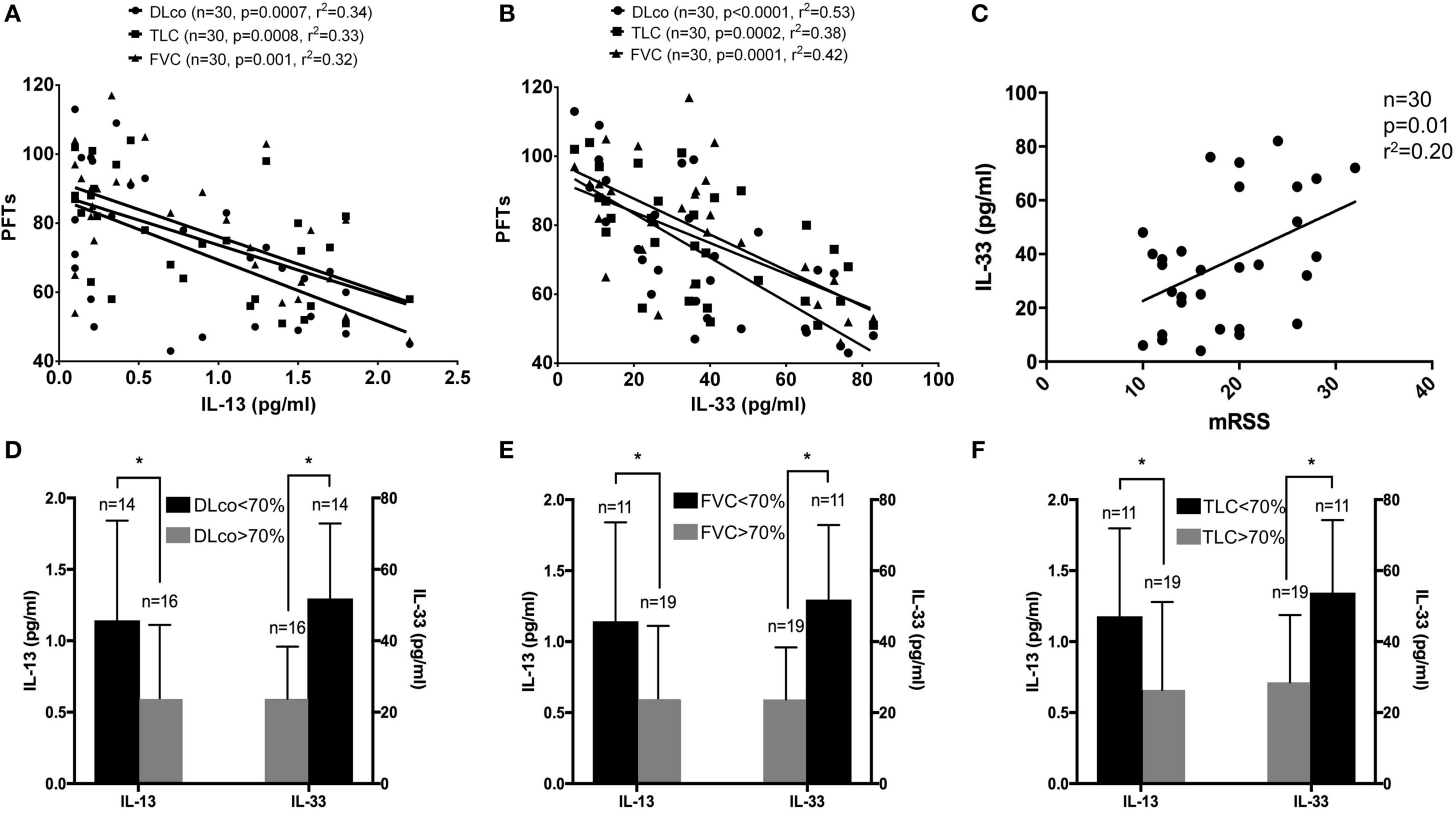
**(A–F)** Linear regression results for IL-13 and IL-33 and pulmonary function tests: both IL-13 and IL-33 levels were significantly associated with DLco (circle), TLC (square) and FVC (triangle) in systemic sclerosis (SSc) patients **(A,B)**. A significant direct association was observed between IL-33 levels and modified Rodnan skin score in the entire cohort of SSc patients **(C)**. IL-13 and IL-33 were plotted, respectively, on the left and right ordinate of each image to show the difference in concentrations according to the 70% cut-off for DLco **(D)**, FVC **(E)** and TLC **(F)** for SSc patients. *p < 0.05.

Next, we wanted to assess whether there was any correlation between both interleukins and pulmonary function test and we observed an inverse correlation between each parameter (DLco, FVC, TLC) and both IL-13 and IL-33 (Figures 1A,B).

Subsequently, we sought to verify the association between the modified Rodnan skin score and IL-13 and IL-33. In this case, we found a significant direct association only with IL-33 levels (Figure 1C), while no significant association was observed for IL-13.

### Interleukins and Interstitial Lung Disease: Subgroup Analysis

As a secondary analysis, we divided our study population according to each pulmonary function parameter in higher than 70% predicted and lower than 70% predicted. As shown in Table 1, Among the entire study population 16 patients had HRCT signs of interstitial lung disease, 14 had a DLco ≤ 70%, and 11 FVC and TLC ≤ 70%. No differences were observed in age, disease and Raynaud's phenomenon durations, mRSS, GI manifestations, scleroderma renal crisis and autoantibodies profile between the groups stratified according to pulmonary function parameters.

Our results showed that both IL-33 and IL-13 are significantly higher in the group of SSc patients with pulmonary function tests lower than 70% (Figures 1D–F). Multiple regression analysis, after adjusting for age, showed that IL-13 and IL-33 are associated with DLco (adjusted *r*^2^ = 0.54, *p* < 0.0001), with FVC (adjusted *r*^2^ = 0.37, *p* < 0.001) and with TLC (adjusted *r*^2^ = 0.42, *p* < 0.0001).

In addition, also Warrick severity score was directly associated with both IL-33 (*r*^2^ = 0.20, *p* = 0.0122) and IL-13 (*r*^2^ = 0.16, *p* = 0.032) when analyzed separately. Multiple regression analysis, after adjusting for age, showed a significant direct association between Warrick severity score and both IL-13 (adjusted *r*^2^ = 0.21, *p* <0.014) and IL-33 (adjusted *r*^2^ = 0.19, *p* <0.022). No relevant association was observed with the Warrick extension score.

## Discussion

The usual natural history of SSc-ILD is characterized by a slow decrease in pulmonary function, with a median survival of 5–8 years (28–30). Although some patients experience a rapid pulmonary decline within the first 3 years of disease, in others ILD represents the initial clinical manifestation of SSc. One of the main unmet needs in SSc clinical trial design is the identification of circulating biomarkers that can accurately serve as predictors of interstitial lung disease progression. Our results show that two interleukins, IL-13 and IL-33, closely related to one another, are increased in a specific subset of systemic sclerosis patients with interstitial lung disease, a relevant complication conferring a high risk for mortality and morbidity.

Indeed, a recent report found that IL-13 levels were associated with the severity of restrictive lung disease in SSc patients with early disease (19, 31), while IL-33 induces migration of Th2 lymphocytes and enhances Th2 cytokine production, such as IL-4, IL-5, and IL-13 *in vitro* (6), thus contributing to the production of collagen by fibroblasts (32) and the activation of pro-inflammatory pathways, such as NF-kappaB. In addition, SSc-ILD has several cellular components involved, including endothelial cells, fibrocytes and fibroblasts and immune cells (33).

Apart from the evidence suggesting the relevance of IL-33 in early SSc, we found that the IL-13/IL-33 axis acquires particular relevance as a marker of disease activity of ILD in SSc patients with the diffuse form, with possible implications for IL-13/IL33 as a future treatment target along with its downstream mediator ST2 (6, 12). IL-33 appears to be one of the main factors that increase early during the disease course in SSc (34), mainly induced by activated endothelial cells (22, 35), thus linking its pro-fibrogenic properties to the vascular disarrangement of the disease (14). Indeed, previous studies reported increased levels of IL-33 in SSc and further support its relevance in the diffuse form of the disease and in subjects with ILD, as observed in our study (19).

This study has some limitations: first of all, the number of patients is too low to support definite conclusions although the patient population has specific inclusion criteria. Secondly, since some SSc patients were under immunosuppressive therapy, its influence on interleukins serum levels cannot be determined. Also, the design of the study and the absence of data regarding disease activity does not allow to draw definite conclusions.

In conclusion, our findings support IL-33 role in association with IL-13 in a subset of SSc patients with ILD and with the diffuse cutaneous form and warrant further longitudinal studies to assess the validity of these two interleukins as potential therapeutic targets and biomarkers for severe SSc-ILD.

## Data Availability Statement

The raw data supporting the conclusions of this article will be made available by the authors, without undue reservation.

## Ethics Statement

The studies involving human participants were reviewed and approved by University of Messina. The patients/participants provided their written informed consent to participate in this study.

## Author Contributions

GB, AV, SG, SCo, AB, WR, and NI contributed to conception and design of the study. CI, CA, DL, TD'A, AC, SCi, and MN organized the database. GB, AB, NI, DL, and SCi performed the statistical analysis. GB, AV, AB, and WR wrote the first draft of the manuscript. SCo, SG, NI, CI, CA, DR, TD'A, AC, and MN wrote sections of the manuscript. All authors contributed to manuscript revision, read, and approved the submitted version.

## Conflict of Interest

The authors declare that the research was conducted in the absence of any commercial or financial relationships that could be construed as a potential conflict of interest.

## Publisher's Note

All claims expressed in this article are solely those of the authors and do not necessarily represent those of their affiliated organizations, or those of the publisher, the editors and the reviewers. Any product that may be evaluated in this article, or claim that may be made by its manufacturer, is not guaranteed or endorsed by the publisher.
